# Hypoxia extends lifespan and neurological function in a mouse model of aging

**DOI:** 10.1371/journal.pbio.3002117

**Published:** 2023-05-23

**Authors:** Robert S. Rogers, Hong Wang, Timothy J. Durham, Jonathan A. Stefely, Norah A. Owiti, Andrew L. Markhard, Lev Sandler, Tsz-Leung To, Vamsi K. Mootha

**Affiliations:** 1 Howard Hughes Medical Institute and Department of Molecular Biology, Massachusetts General Hospital, Boston, Massachusetts, United States of America; 2 Broad Institute, Cambridge, Massachusetts, United States of America; 3 Department of Systems Biology, Harvard Medical School, Boston, Massachusetts, United States of America; 4 Department of Biological Engineering, Massachusetts Institute of Technology, Cambridge, Massachusetts, United States of America; Buck Institute for Research on Aging, UNITED STATES

## Abstract

There is widespread interest in identifying interventions that extend healthy lifespan. Chronic continuous hypoxia delays the onset of replicative senescence in cultured cells and extends lifespan in yeast, nematodes, and fruit flies. Here, we asked whether chronic continuous hypoxia is beneficial in mammalian aging. We utilized the *Ercc1 Δ/-* mouse model of accelerated aging given that these mice are born developmentally normal but exhibit anatomic, physiological, and biochemical features of aging across multiple organs. Importantly, they exhibit a shortened lifespan that is extended by dietary restriction, the most potent aging intervention across many organisms. We report that chronic continuous 11% oxygen commenced at 4 weeks of age extends lifespan by 50% and delays the onset of neurological debility in *Ercc1 Δ/-* mice. Chronic continuous hypoxia did not impact food intake and did not significantly affect markers of DNA damage or senescence, suggesting that hypoxia did not simply alleviate the proximal effects of the *Ercc1* mutation, but rather acted downstream via unknown mechanisms. To the best of our knowledge, this is the first study to demonstrate that “oxygen restriction” can extend lifespan in a mammalian model of aging.

## Abstract 2

Aging is one of the strong risk factors for the most common diseases on our planet, but we have few interventions that delay aging (including dietary restriction). This study reveals that a different type of restriction - oxygen restriction - can extend lifespan and counter neurological demise in a mouse model of accelerated aging.

## Introduction

Aging is a major risk factor for the most common human diseases including cancer, cardiovascular disease, and neurodegeneration [1]. Several key “hallmarks of aging” have been defined [2], which has stimulated progress on finding interventions to counteract the aging process. The National Institute of Aging Interventions Testing Program has identified 6 compounds (rapamycin, acarbose, 17-α-estradiol, the Nrf2-activator Protandim, the anti-oxidant nordihydroguaiaretic acid, and aspirin) that extend lifespan in HET3 mice (the genetically heterogenous offspring of a four-way cross of laboratory mouse strains) [3]. These compounds target central regulators of cellular homeostasis such as mTOR, SIRT1, and Nrf2 to modulate nutrient sensing, oxidative stress, and inflammation [4]. Work on aging interventions has matured to the point where metformin, a widely prescribed diabetes medication that targets mitochondrial complex I, is now being tested in human clinical trials as an antiaging intervention in older adults with the end-point being a composite score of the onset of the most common age-related chronic diseases and death [5].

Among the list of interventions that slow aging, dietary restriction stands out both for its effect size and the number of species in which it has been proven effective [6]. Dietary restriction significantly extends lifespan in yeast, roundworms, fruit flies, mice, and rats [6]. Despite being first reported in 1935 [7], a full understanding of the therapeutic mechanisms of dietary restriction remains elusive, as no single genetic or pharmacologic intervention (or combination of such interventions) is sufficient to fully recapitulate its effects [8]. Decades of studies have shown that dietary restriction has many complex effects and influences expression of several hundred genes across multiple tissues [9] through its integrated effects on the growth hormone/insulin, mTOR, and sirtuin signaling pathways [6].

Another type of restriction, “oxygen restriction,” or continuous hypoxia (to varying degrees depending on the organism), has also been reported to delay senescence and aging in cellular and animal models. Hypoxia significantly delays the onset of replicative senescence in cultured mammalian cells. Compared to standard atmospheric conditions (21% oxygen at sea level), hypoxia extends the number of population doublings until replicative senescence in mouse embryonic fibroblasts [10], primary human lung fibroblasts [11], and even in the presence of specific senescence-inducers such as etoposide and nutlin-3a [12]. In *Saccharomyces cerevisiae*, hypoxia induced by limited culture aeration extends chronological lifespan, and in fact, merely 2 days of early growth under hypoxic conditions is sufficient to increase survival at 3 weeks [13]. In *Caenorhabditis elegans*, 0.5% oxygen introduced at the L4 larval stage increases median lifespan by over 12% in a manner dependent upon *hif1-a* and *daf-16* [14]. In *Drosophila melanogaster*, provided the juveniles are reared in 21% oxygen, 10% oxygen optimizes adult median and maximal lifespan [15], whereas, hyperoxia hastens death and neurodegeneration [15,16].

While the above studies come from cell culture and invertebrate models, 2 observations raise the possibility that hypoxia could slow mammalian aging. First, the naked mole rat (*H*. *glaber*), whose lifespan far exceeds that which would be predicted by phylogeny or body mass, experiences significant durations of relative ambient hypoxia because of extreme crowding in their burrows (though the precise oxygen tension has not been measured in their natural environment [17]). Second, in genetically heterogenous HET3 mice, a hypoxia transcriptomic signature appears to be shared among myriad interventions shown to extend lifespan in both the NIA Interventions Testing Program and long-lived mutants [3].

A natural question is therefore whether oxygen restriction, like dietary restriction, may be beneficial in mammalian aging. Here, we explore this question using the *Ercc1 Δ/-* mouse model of accelerated aging. *Ercc1* is essential for the nucleotide excision repair (NER) pathway of DNA damage repair [18], and accumulation of DNA damage is a “hallmark” [2] of aging which is tightly coupled to variation in species’ lifespans [19]. The *Ercc1 Δ/-* mouse is a particularly useful model of accelerated aging because it exhibits a shortened lifespan of less than 6 months and early onset of anatomic, physiological, and molecular features of advanced age across multiple tissues [18,20–22]. Moreover, prior work has shown that interventions that extend lifespan and healthspan in multiple wild-type organisms—notably dietary restriction [23], but to some extent also rapamycin [24], and senolytics [25]—also confer benefit to the *Ercc1 Δ/-* phenotype. Here, we report that chronic continuous hypoxia—“oxygen restriction”—extends lifespan and delays neurologic debility in this model.

## Results

### Effect of hypoxia on survival and neurologic function of *Ercc1 Δ/-* mice

*Ercc1 Δ/-* mice are born without overt developmental defects but begin to show motor deficits in clasping by 5 weeks [21]. We initiated hypoxia therapy at the time of weaning at 4 weeks of age. We transferred mice to a normobaric chamber with 11% oxygen achieved through dilution of air with nitrogen.

**Fig 1:**
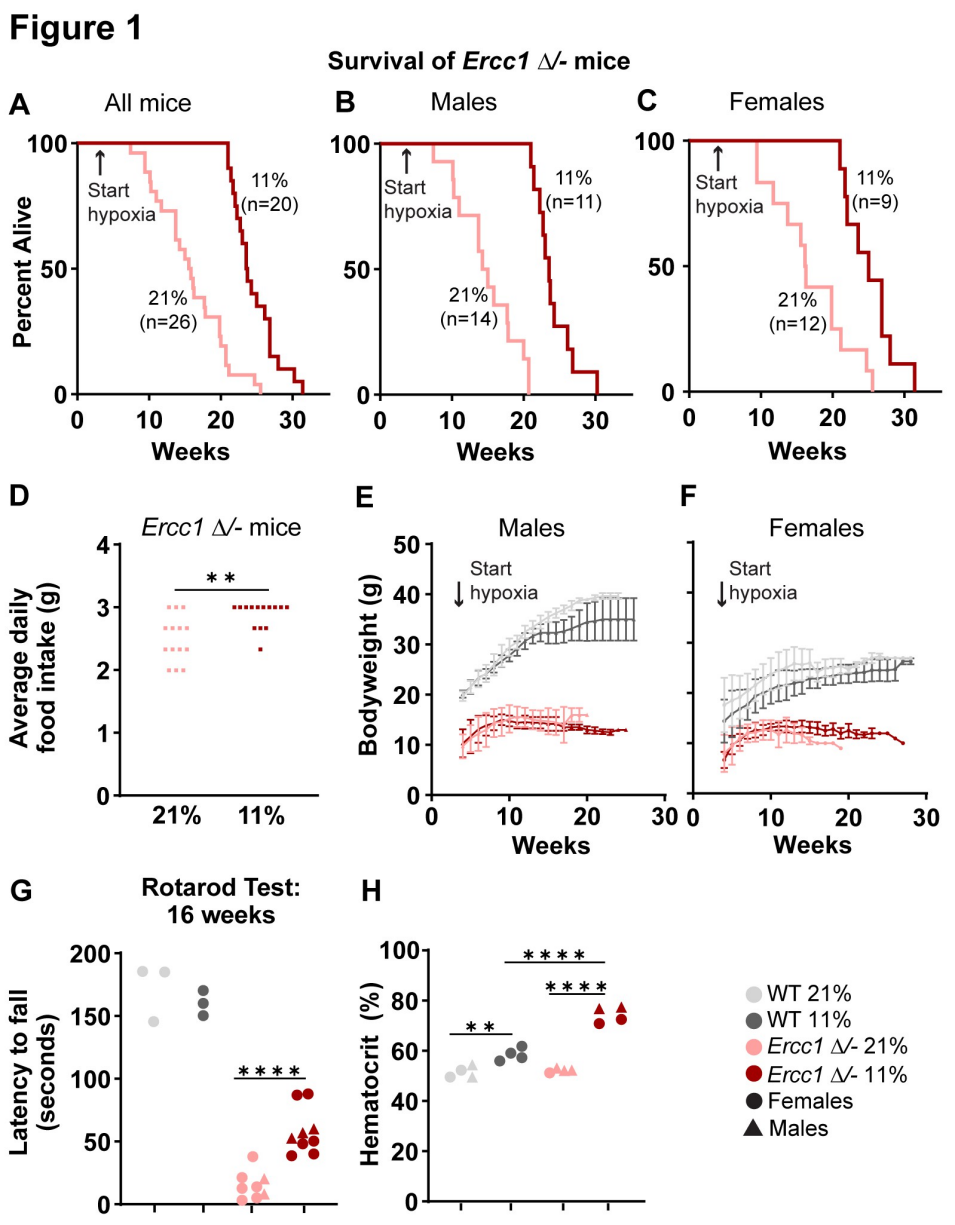
The effect of hypoxia on survival, neurologic function, body weight, and hematocrit. **(A–C)** Survival of *Ercc1 Δ/-* mice maintained at 21% (14 males, 12 females) vs. 11% (11 males, 9 females) oxygen. Mantel–Cox *P* < 0.01 for median survival in all comparisons. S1 Data, Worksheet “Survival”. **(D)** Average daily food intake for 3 mice (2 females, 1 male) for 14 consecutive days between weeks 8 and 12 of age; food intake quantified at the cage level, thus the use of squares. Two-tailed t test. S1 Data, Worksheet “Food&WaterIntake”. **(E)** Body weight: males, *n* = 4 WT at 21% oxygen, 6 WT at 11% oxygen, 12 *Ercc1 Δ/-* at 21% oxygen, 9 *Ercc1 Δ/-* at 11% oxygen. **(F)** Body weight: females, *n* = 9 WT at 21% oxygen, 7 WT at 11% oxygen, 6 *Ercc1 Δ/-* at 21% oxygen, 6 *Ercc1 Δ/-* at 11% oxygen. Data plotted as mean ± standard deviation. S1 Data, Worksheet “Bodyweights”. **(G)** Accelerating rotarod test at 16 weeks of age (*n* = 3 per WT group, *n* = 8–9 per *Ercc1 Δ/-* group). Dunnett’s multiple comparison tests. S1 Data, Worksheet “Rotarod.” **(H)** Hematocrit at 12 weeks of age (*n* = 4 per group). Tukey’s multiple comparison tests. S1 Data, Worksheet “Hematocrit.” **** = *p* < 0.0001; *** = *p* < 0.001; ** = *p* < 0.01; * = *p* < 0.05; ns = not significant.

Continuous hypoxia extended median lifespan of *Ercc1 Δ/-* mice by 50% (Fig 1A) (23.6 weeks versus 15.7 weeks, Mantel–Cox *P* < 0.0001, *n* = 20 in hypoxia; *n* = 26 in normoxia) and maximum lifespan from 25.6 to 31.4 weeks (Wang–Allison *P* < 0.01). The effect size was similar between males (Fig 1B) (median: 23.6 weeks versus 14.7 weeks, *P* < 0.0001, *n* = 11 in hypoxia; *n* = 14 in normoxia; maximum: 30.3 weeks versus 20.7 weeks, *P* = 0.06) and females (Fig 1C) (median: 25.0 weeks versus 16.2 weeks, *P* < 0.01, *n* = 9 in hypoxia; *n* = 12 in normoxia; maximum: 31.4 weeks versus 25.6 weeks, *P* = 0.01).

Given that dietary restriction is the single most effective intervention to increase lifespan and healthspan of *Ercc1 Δ/-* mice [23], we sought to determine whether food intake was impacted by hypoxia. Importantly, hypoxia did not induce dietary restriction in *Ercc1 Δ/-* mice. Daily food intake was measured for 14 consecutive days between weeks 8 and 12 of life, and *Ercc1 Δ/-* mice in hypoxia actually consumed more food than those in normoxia (Fig 1D) (2.9 grams versus 2.5 grams of food per mouse per day, *P <* 0.01, *n* = 3 per group). Furthermore, while *Ercc1 Δ/-* mice are substantially smaller than wild-type mice at all time points and fail to gain weight normally, the body weights of *Ercc1 Δ/-* mice in hypoxia and normoxia did not differ (Fig 1E and 1F). By contrast, *Ercc1 Δ/-* mice treated with dietary restriction, despite their improved health relative to *Ercc1 Δ/-* mice fed ad libitum, are even smaller than *Ercc1 Δ/-* mice fed ad libitum [23].

Concordant with the extension of lifespan, we also observed improvement in motor function in *Ercc1 Δ/-* mice in hypoxia. At 16 weeks of age, *Ercc1 Δ/-* mice maintained in normoxia are substantially debilitated, whereas those maintained in hypoxia perform the accelerating rotarod test significantly better (Fig 1G) (58.0 s versus 15.4 s, *P* < 0.0001, *n* = 9 in hypoxia; *n* = 8 in normoxia).

As expected, wild-type mice in continuous hypoxia had a significant increase in hematocrit measured at 12 weeks (Fig 1H) (51.5% versus 58.6%, *P* < 0.01, *n* = 4 per group), roughly similar to the increases reported in mice after exposure to chronic sustained hypoxia of similar magnitude for several weeks [26,27]. Curiously, in *Ercc1 Δ/-* mice the increased hematocrit was even more pronounced (52.3% versus 74.4%, *P* < 0.0001; *n* = 4 per group). This was accompanied by greater reticulocytosis (1.2% versus 1.9%, *P* < 0.01), implying a differential erythropoietic response to hypoxia in *Ercc1 Δ/-* mice, although a dehydration effect is also possible, as daily water intake measured between weeks 8 and 12 of life was modestly lower as well (2.3 mL versus 1.9 mL per mouse per day, *P* < 0.01, *n* = 3 per group). At present, we do not know the mechanistic basis for the exuberant polycythemia in response to hypoxia in *Ercc1 Δ/-* mice, but it is noteworthy, because if the therapeutic mechanism of hypoxia is dependent on achieving decreased brain oxygen tension, as it is for mouse models of mitochondrial neurological disease [28], a compensatory increase in hematocrit that augments brain oxygen delivery might blunt the beneficial effect of hypoxia over time.

### Evaluation of candidate mechanisms of the therapeutic effect of hypoxia in *Ercc1 Δ/-* mice

Having observed that hypoxia significantly extended lifespan and delayed neurologic debility, we next sought insight on the potential mechanism(s) of this therapeutic effect. The current model of *Ercc1 Δ/-* pathophysiology posits that impaired NER leads to the accumulation of DNA damage; the ensuing DNA damage response causes accelerated cellular senescence and organ dysfunction which is further amplified by the senescence-associated secretory proteome (SASP) [20]. Interventions demonstrated to ameliorate the *Ercc1 Δ/-* phenotype—dietary restriction [23], rapamycin [24,29], the senolytic fisetin [25]—have been accompanied by improvements in parameters along this presumed causal genetic pathway.

We first considered whether hypoxia affected the accumulation of DNA damage broadly throughout the organism using the widely established DNA damage marker γH2Ax [30]. We performed immunohistochemistry in kidney, liver, spleen, and heart of mice at approximately 15 weeks of age. As expected [23], we found a significant increase in γH2Ax-positive cells in *Ercc1 Δ/-* mice relative to wild type in liver and kidney (S1A Fig). However, hypoxia did not attenuate the increase in this marker of DNA damage. Spleens had the highest percentage of γH2Ax-positive cells across all organs, while in the heart, γH2Ax-positive cells were very sparse (0–1 positive cell per mm^2 of tissue). We then interrogated whether hypoxia modulated the induction of cell cycle arrest and senescence in the vital organs where it has been reported in *Ercc1 Δ/-* mice, kidney and liver, reflected by increased expression of *Cdkn1a* (p21) and *Cdkn2a* (p16) assessed with qPCR [20]. Again, we observed large increases in the expression of these senescence markers in kidney and liver of *Ercc1 Δ/-* mice relative to wild type; however, levels of *Cdkn2a* were unaffected by hypoxia (S1B Fig), and although levels of *Cdkn1a* trended slightly lower in hypoxia (S1C Fig), the difference did not reach statistical significance.

We next focused on the brain, and in particular the cerebellum, for 3 reasons. First, while *Ercc1 Δ/-* mice have accelerated multiorgan degenerative changes, the proximate cause of death is likely neurologic, as ataxia and incoordination are early and prominent features [21], and loss of Purkinje neurons are a manifestation of *Ercc1 Δ/-* pathology [23,29]. Second, while no tissue-specific knockout of *Ercc1* fully recapitulates the global knockout, Purkinje-specific knockout causes severe neurological impairment [31]. Third, prior work in our laboratory focused on global knockout mutant mice with multiorgan pathologies has shown the brain to be particularly responsive to the therapeutic effect of hypoxia [32,33].

**Fig 2:**
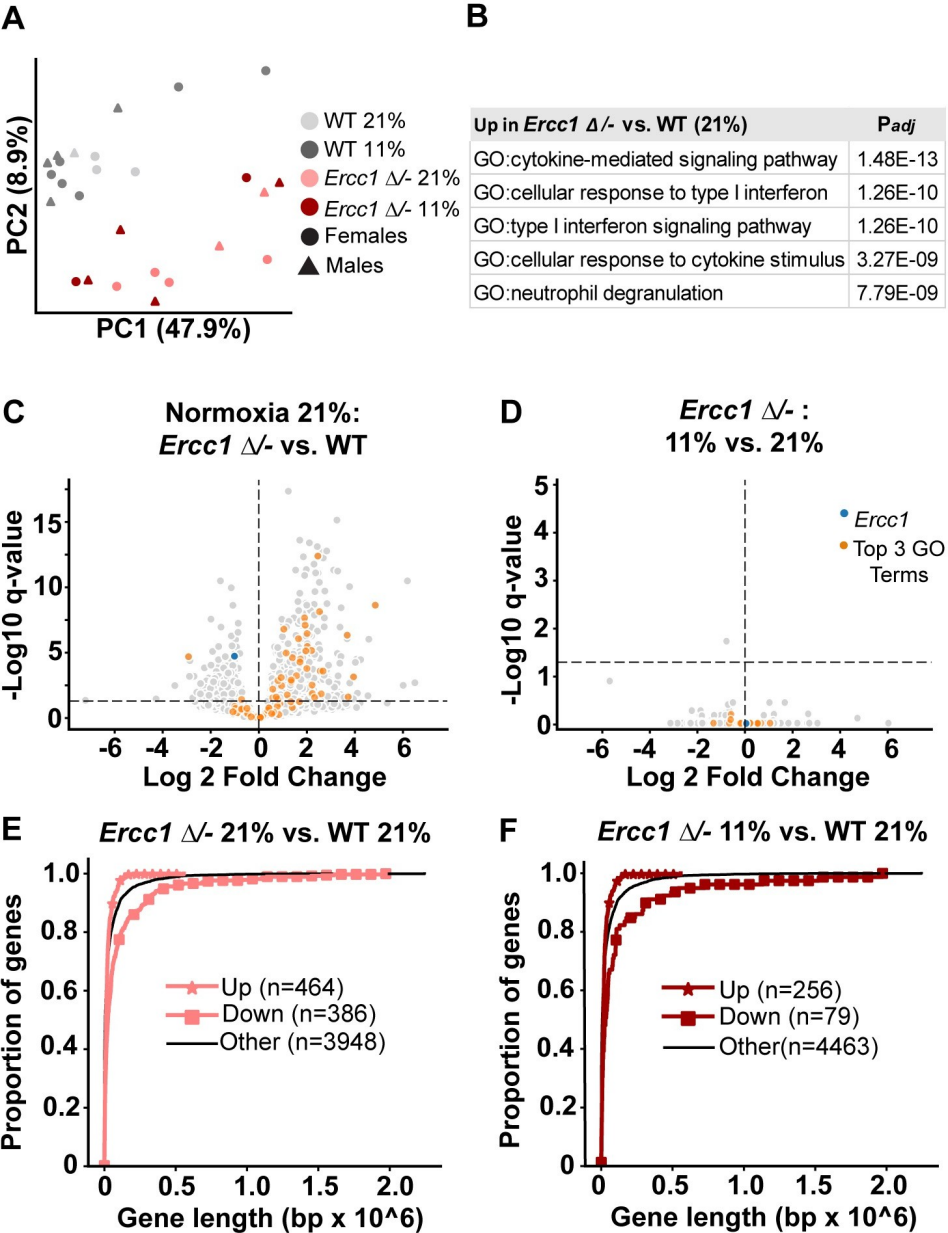
The effect of hypoxia on the cerebellar transcriptome. **(A)** Principal component analysis. **(B)** The top 5 GO terms most significantly associated with the 223 genes increased in *Ercc1 Δ/-* (21%) vs. WT (21%) with Log2FC > 1.25 and FDR < 0.01; further details in S3 Table. **(C)** Volcano plot in Normoxia (21%): *Ercc1 Δ/-* vs. WT. **(D)** Volcano plot of *Ercc1 Δ/-*: Hypoxia (11%) vs. Normoxia (21%). Genes colored orange comprise the union of genes listed in the Top 3 GO Terms (see S3 Table) = “cytokine-mediated signaling pathway GO:0019221”; “cellular response to type I interferon (GO:0071357)”; “type I interferon signaling pathway (GO:0060337)”. **(E, F)** Cumulative distribution functions of cerebellar gene expression by gene length, a marker of transcriptional stress. For A–F, (*N* = 26; *n* = 4 WT normoxia, *n* = 10 WT hypoxia, *n* = 6 *Ercc1 Δ/-* normoxia, *n* = 6 *Ercc1 Δ/-* hypoxia at 15–19 weeks of age). Mouse basic metadata in S1 Data, Worksheet “RNAseq”.

To gain a broad understanding of the state of the mutant and wild-type cerebellum under hypoxia and normoxia, we generated bulk RNA-seq data at the time when *Ercc1 Δ/-* normoxic mice of a given cohort reached euthanasia criteria, ranging between 15 and 19 weeks. We analyzed differential gene expression to spotlight pathways potentially altered in the *Ercc1 Δ/-* brains and/or impacted by oxygen. We considered 4,798 genes that met a minimum threshold (see Materials and methods) for mean level of expression and dispersion. *Ercc1 Δ/-* and wild-type mice clearly separated along Principal Component 2 (Fig 2A), but no principal component separated samples by oxygen status. Using a log2-fold change threshold of 1.25 and a false discovery rate (FDR) of 1%, we identified 223 genes with increased expression in *Ercc1 Δ/-* versus wild-type mice under conditions of normoxia (S1 Table) and 80 genes with decreased expression (S2 Table). The dominant signature of the genes with increased expression in *Ercc1 Δ/-* cerebella was that of neuroinflammation and innate immune activation; of the top 50 GO terms associated with this list (Fig 2B and 2C and S3 Table), manual inspection reveals that all pertain to immune and complement activation, consistent with *Ercc1 Δ/-* mice experiencing significant neurodegenerative changes by 4 months of age [21]. We next compared gene expression between *Ercc1 Δ/-* mice maintained in hypoxia versus *Ercc1 Δ/-* mice maintained in normoxia. Surprisingly, despite the far more robust phenotype of the *Ercc1 Δ/-* mice in hypoxia at this stage, there was only 1 gene (*Armcx5*) and no GO pathways with significantly different expression in hypoxia versus normoxia (Fig 2D).

In addition to providing insight on differential gene expression, previous studies have shown that transcriptomic data can measure global DNA damage in *Ercc1 Δ/-* mice (“transcriptional stress”), whereby longer genes are more likely to have decreased expression, because *Ercc1* is essential for transcription-coupled NER, and the probability of endogenous DNA damage increases with gene length [29]. We analyzed differential gene expression as a function of gene length in our cerebellar RNA-seq data. Consistent with previous studies [29], we observed that in normoxia (Fig 2E), shorter genes were significantly enriched among the genes with increased expression in *Ercc1 Δ/-* relative to wild type (Kolmogorov–Smirnov *D-*statistic = 0.08, *P* = 0.003), whereas longer genes (which are more prone to DNA damage purely because of their length) were significantly enriched among the genes with decreased expression (*D-*statistic: 0.28, *P* = 1.4 × 10^−24^). This pattern of transcriptional stress persisted in *Ercc1 Δ/-* mice in hypoxia (Fig 2F; increased genes: *D-*statistic: 0.09, *P* = 0.02; decreased genes: *D-*statistic: 0.29, *P* = 2.3 × 10^−6^), suggesting that hypoxia did not attenuate transcriptional stress in *Ercc1 Δ/-* cerebella.

Overall, despite the fact that *Ercc1 Δ/-* mice maintained in hypoxia are significantly more robust at approximately 4 months of age than those maintained in normoxia, we did not observe this difference reflected in the cerebellar transcriptome nor in classic markers of DNA damage and senescence in peripheral tissues.

Finally, we considered whether the brains of *Ercc1 Δ/-* mice exhibit perturbations of mitochondrial homeostasis, which if present, could potentially be alleviated by hypoxia. Although *Ercc1 Δ/-* mice exhibit many features of accelerated aging and mitochondrial dysfunction is a hallmark of aging [2], the effect of *Ercc1* deficiency on mitochondrial function has not been previously explored in detail. Additionally, our prior work had shown the therapeutic benefit of chronic continuous hypoxia specifically in models of mitochondrial disease [32–34]. We therefore performed sequencing of the entire 16.6 kb mtDNA molecule from mouse forebrains at 15 to 19 weeks of age and assessed for the presence of single-nucleotide variants (SNVs), which are known to accumulate in aged mice [35]. Interestingly, neither the *Ercc1 Δ/-* mutation nor hypoxia caused any increase in SNV accumulation; across all groups, we observed no mice with an SNV with heteroplasmy greater than 2%. Consistent with this finding, there was no consistent pattern of differential expression of the 13 mitochondrially-encoded mt-mRNAs (S1D Fig), nuclear encoded electron transport chain (ETC) genes (S1D Fig) or ETC proteins (S1E Fig) in *Ercc1 Δ/-* mice brains.

## Discussion

To the best of our knowledge, the current study is the first to report that hypoxia extends lifespan in a mouse model of aging. We have demonstrated that continuous hypoxia (11% oxygen)—or “oxygen restriction”—significantly extends lifespan of *Ercc1 Δ/-* mice and delays neurologic morbidity. In this model, hypoxia appears to be the second strongest intervention to date, second only to dietary restriction [23]. Our findings add to a nascent but burgeoning literature on the beneficial effect of hypoxia in a wide variety of neurologic disease models. Chronic continuous hypoxia has been reported as beneficial in at least 3 other mouse models of neurologic disease. In 2 mitochondrial disease models, hypoxia corrects defects that arise as a consequence of the genetic lesion—decreasing excessive molecular oxygen in the setting of a defective ETC in *Ndufs4* knockout [28] and restoring iron sulfur cluster levels in *frataxin* knockout [32]. In the experimental autoimmune encephalitis model of multiple sclerosis, continuous 10% oxygen promotes vascular integrity and apoptosis of infiltrating leukocytes [36]. The ability of hypoxia to alleviate brain degeneration in such diverse models points either to the pleiotropic effects of oxygen restriction, or alternatively, the existence of a downstream and convergent neuroprotective mechanism.

An important future goal is to define the mechanism by which chronic continuous hypoxia is extending lifespan in this model, and the extent to which this mechanism overlaps with that of pathways known to be involved in aging, such as mTOR and insulin signaling. Three plausible mechanisms are the following: (i) activation of the HIF pathway; (ii) diminution of oxidative stress; and (iii) interruption of the vicious cycle of neurodegeneration and neuroinflammation. With respect to HIF pathway activation, in our prior work in the *Ndufs4* KO model, we showed that HIF activation was not sufficient to recapitulate the benefits of hypoxia [28], and in the current work, we did not detect a signature of HIF activation in the brain based on RNA-seq. With respect to diminution of oxidative stress, given the basal level of oxidative damage to DNA, one might expect to observe a beneficial effect of hypoxia on the accumulation of DNA damage in a mutant with severe defects in DNA damage repair. Our RNA-seq studies did not find evidence of hypoxia attenuating the previously reported transcriptional stress that is believed to be associated with DNA damage in *Ercc1 Δ/-* mice; however, this is a relatively indirect measure of DNA damage and more sensitive assays [37] might reveal an effect of hypoxia on specific manifestations of defective NER, such as the accumulation of cyclopurines and other bulky DNA adducts [38]. It must be noted that in addition to potentially influencing the level of oxidative damage to key cellular structures from ROS, hypoxia modulates ROS signaling more broadly. In multiple contexts, hypoxia has been demonstrated to increased lifespan (yeast [39], *C*. *elegans* [40,41]) or time to replicative senescence (primary human lung fibroblasts [11]), via an increase in ROS production which then activates life-extending pathways, a form of hormesis [42]. Future work will be required to carefully assess whether hypoxia increases or decreases ROS accumulation in *Ercc1 Δ/-* tissues and whether the net effect of any change in ROS levels is beneficial or deleterious. Lastly, it is notable that 3 mouse models of brain disease that are alleviated with hypoxia—*Ndufs4* KO [43], experimental autoimmune encephalomyelitis [36], and *Ercc1 Δ/-*—demonstrate severe neuroinflammation as a major feature of their pathology. Prior work in *Ercc1 Δ/-* brains and spinal cords has demonstrated progressive microglial activation [21]. Neuroinflammation is believed to amplify neurodegeneration, thus establishing a vicious cycle [44]. At present, we do not know where in this vicious cycle between neuronal damage and inflammation hypoxia exerts its effect—through dampening the inflammatory response to neuronal injury, or conferring neuronal resilience to the stress of DNA damage and inflammation, or some combination of the two. In either case, the vicious cycle appears to be blunted.

In addition to defining the molecular mechanism of chronic continuous hypoxia, future work must determine if this mechanism operates powerfully across all organs, or rather, if its effects are most notable in the brain. In models of mitochondrial disease, the therapeutic effect of hypoxia is most profound in the brain [28,32,33]. The proximate cause of death in *Ercc1 Δ/-* mice is neurologic debility and neurologic dysfunction is an early and prominent feature of the phenotype [21]. However, it is not clear that neurodegeneration accounts for all of the neurologic debility, as opposed to encephalopathy from renal and hepatic dysfunction [45]. Tissue-specific *Ercc1* knockout in Purkinje [31], excitatory forebrain [46], and microglial cells [47] fail to fully recapitulate the whole-body *Ercc1 Δ/-* phenotype. Therefore, to determine if hypoxia promotes neuronal resilience in a cell autonomous manner, it would be useful to subject these neuron-specific mutants to our hypoxia regimen. Future studies should also further characterize the extent to which different neurological functions—object recognition, strength, coordination, sensation—are preserved by hypoxia using a broader battery of neurologic tests.

To understand more fully the therapeutic potential of chronic continuous hypoxia in this aging model, we must also learn if in comparison to the benefits of dietary restriction its benefits are additive, synergistic, or antagonistic. If, for example, the therapeutic mechanism of chronic continuous hypoxia involves improved insulin sensitivity (stabilization of HIF2α in liver improves insulin sensitivity [48] and dietary restriction in *Ercc1 Δ/-* mice also improves insulin sensitivity [23]), one would not expect significant additive benefit of hypoxia with dietary restriction. By contrast, if (as it appears) hypoxia confers neuronal resilience through a novel mechanism, it might have additive benefit on top of dietary restriction. Studies testing these 2 interventions simultaneously are required to address this important question.

Dietary restriction is of great scientific interest because it is effective across multiple preclinical models. Importantly, some of the benefits of full dietary restriction can be achieved with regimens less arduous than a full 30% reduction in baseline caloric intake, such as single meal feeding, which imposes a prolonged daily fasting interval [49]. Similarly, it will also be important to determine whether more practical hypoxia regimens, such as intermittent hypoxia, or a more moderate degree of hypoxia (e.g., 17% oxygen, equivalent to the effective oxygen tension in Denver) are effective. Although repeated acute intermittent hypoxia has been used to therapeutic effect in the C2 hemi-section model of spinal cord injury [50], intermittent hypoxia (10 h 11% oxygen, 14 h 21% oxygen) was not beneficial in the *Ndufs4* KO mouse model of Leigh syndrome [34].

In summary, this is the first study to demonstrate that “oxygen restriction,” analogous to “dietary restriction,” can extend lifespan in a mammalian model of aging. As with all models of aging, it remains to be seen whether and to what extent the findings of this model generalize to wild-type aging. There might be fundamental aspects of *Ercc1 Δ/-* physiology that limit their generalizability; for example, rapamycin fails to extend *Ercc1 Δ/-* lifespan despite doing so in wild-type mice, and we did not observe the well-established inhibitory effect of hypoxia on food intake and weight gain that is seen in wild-type mice in *Ercc1 Δ/-* mice. It is conceivable that for hypoxia to extend lifespan, it needs to be started at a young age, as 10% oxygen begun at the advanced age of 21 months hastens wild-type mouse death secondary to pulmonary hypertension [51].

Epidemiologic evidence suggests that lifelong oxygen restriction might slow the aging process in humans. Though there are many potential confounders to this finding, recent cross-sectional studies in Bolivia have demonstrated significant enrichment for nonagenarians and centenarians at very high altitudes [52]. There is also intriguing data that suggests there are potential benefits of moving to altitude in adulthood. In a longitudinal study of over 20,000 soldiers of the Indian Army assigned to serve at 2 to 3 mile elevations above sea level for 3 years between 1965 and 1972, their risk of developing the major sources of age-related morbidity in modern societies—diabetes mellitus, hypertension, and ischemic heart disease—was a fraction of the risk of their comrades serving at sea level [53]. Our initial findings establish oxygen restriction as a potential aging intervention, motivating the search for underlying mechanisms and generalizability to other mammalian models.

## Materials and methods

### Ethics statement

All mouse studies were approved by the Subcommittee on Research Animal Care and the Institutional Animal Care and Use Committee of Massachusetts General Hospital under Protocol #2011N000077.

C57BL/6 *Ercc1 +/-* and FVB *Ercc1 +/Δ* mice were generously provided by the laboratory of Dr. Ingrid van der Pluijm and bred as initially described by Weeda and colleagues in 1997 [18]. All experiments were performed at sea level. For hypoxia (11% oxygen), mice were placed in BioSpherix OxyCycler Model A84XOV (Parish, New York) to achieve dilution of air with nitrogen. Details of histological preparation and analysis for quantification of hematocrit, reticulocytes, γH2Ax-positive cells, and tissue preparation for qPCR and western immunoblots is provided in S1 Extended Methods. RNA-seq was performed with the Illumina HiSeq 4000 platform (GeneWiz, now Azenta, South Plainfield, New Jersey), and mtDNA sequencing was performed with the Illumina MiSeq platform. For differential gene expression, statistical analyses were performed using the DESeq2 package with the threshold for significance applied as indicated in the text. The Wang–Allison test (two-tailed to be conservative) of maximal survival, cumulative density functions, and tests of differential ETC gene expression were calculated in Python [54,55]. Further details are provided in S1 Extended Methods.

## Supporting information

S1 Data **Contains several worksheets with data relevant to the specific figures as indicated:** “Survival”–Fig 1A–1C. “Food&WaterIntake”–Fig 1D. “BodyWeights”–Fig 1E and 1F. “Rotarod”–Fig 1G. “Hematocrit”–Fig 1H; “Reticulocytes”–Results section paragraph 5. “RNAseq”–Basic metadata for mice used in RNAseq studies relevant to Figs 2A–2F and S1D. Complete metadata and raw data has been deposited in GEO (GSE219203) and SRA (PRJNA910556). Code can be accessed at https://github.com/MoothaLab/ercc1-mouse-rob. “gammah2xIHC”–S1A Fig; for representative images, S2 Fig. “qPCR”–S1B and S1C Fig. “mtDNA”–Basic metadata for mice used in mtDNA sequencing studies. Complete metadata and raw data has been deposited in GEO (GSE219203) and SRA (PRJNA910556). Code can be accessed at https://github.com/MoothaLab/ercc1-mouse-rob. “westerns”–Basic metadata for mice used in S1E Fig.
(XLSX)

S1 Fig The effect of *Ercc1* deficiency and hypoxia on peripheral markers of DNA damage and senescence and the expression of OXPHOS genes in brain. **(A)** Quantification of cells with ɤH2Ax foci at approximately 15 weeks of age (*n* = 3 per WT group, *n* = 6 per *Ercc1 Δ/-* group). Dunnett’s multiple comparisons test. S1 Data, Worksheet “gammaH2xIHC”; representative images in S2 Fig. **(B, C)** Relative gene expression of senescence markers at 14–19 weeks of age (*n* = 5–6 per group—dots plotted as mean of 2 technical replicates per sample, statistics calculated as nested analyses). Dunnett’s multiple comparisons test. S1 Data, Worksheet “qPCR”. **(D)** Relative expression of the 13 mt-mRNAs and 5 representative nuclear-encoded mRNAs in cerebellum at 15–19 weeks; *n* = 4, 10, 6, 6 as in Fig 2A–2F. Mouse basic metadata in S1 Data, Worksheet “RNAseq”. **(E)** Western blot of ETC subunits in forebrain at 14 to 15 weeks with accompanying beta-actin loading control and Coomassie blue gel stain; *n* = 3 per group. RH = purified rat heart mitochondria (positive control). Mouse metadata in S1 Data, Worksheet “westerns”. **** = *p* < 0.0001; *** = *p* < 0.001; ** = *p* < 0.01; * = *p* < 0.05; ns = not significant.
(TIF)

S2 Fig Contains representative images of histology quantified in S1A Fig. (PDF)

S1 Table Lists the 223 genes increased in Ercc1 Δ/- vs. WT in normoxia (21%) with log2-Fold Change > 1.25 and adjusted *p*-valued <0.01. (XLSX)

S2 Table Lists the 80 genes decreased in Ercc1 Δ/- vs. WT under normoxia (21%) with absolute value of the log2-Fold Change >1.25 and adjusted *p*-value <0.01. (XLSX)

S3 Table Lists the top 50 GO terms associated with the 223 genes listed in S1 Table. Note, there were no GO terms associated with the 80 genes listed in S2 Table with adjusted *p*-value <0.01.
(XLSX)

S1 Extended Methods Contains detailed Materials and methods used and accompanying citations. (DOCX)

S1 Raw Images Contains original uncropped blot and gel results for S1E Fig. (PDF)

## Abbreviations

ETC: electron transport chain
FDR: false discovery rate
NER: nucleotide excision repair
SASP: senescence associated secretory proteome
SNV: single-nucleotide variant
