# Supplementary figures and images for: Hypoxia extends lifespan and neurological function in a mouse model of aging
Source: PLoS Biol. 2023 May 23;21(5):e3002117. doi: 10.1371/journal.pbio.3002117 (PMC10204955; doi:10.1371/journal.pbio.3002117)

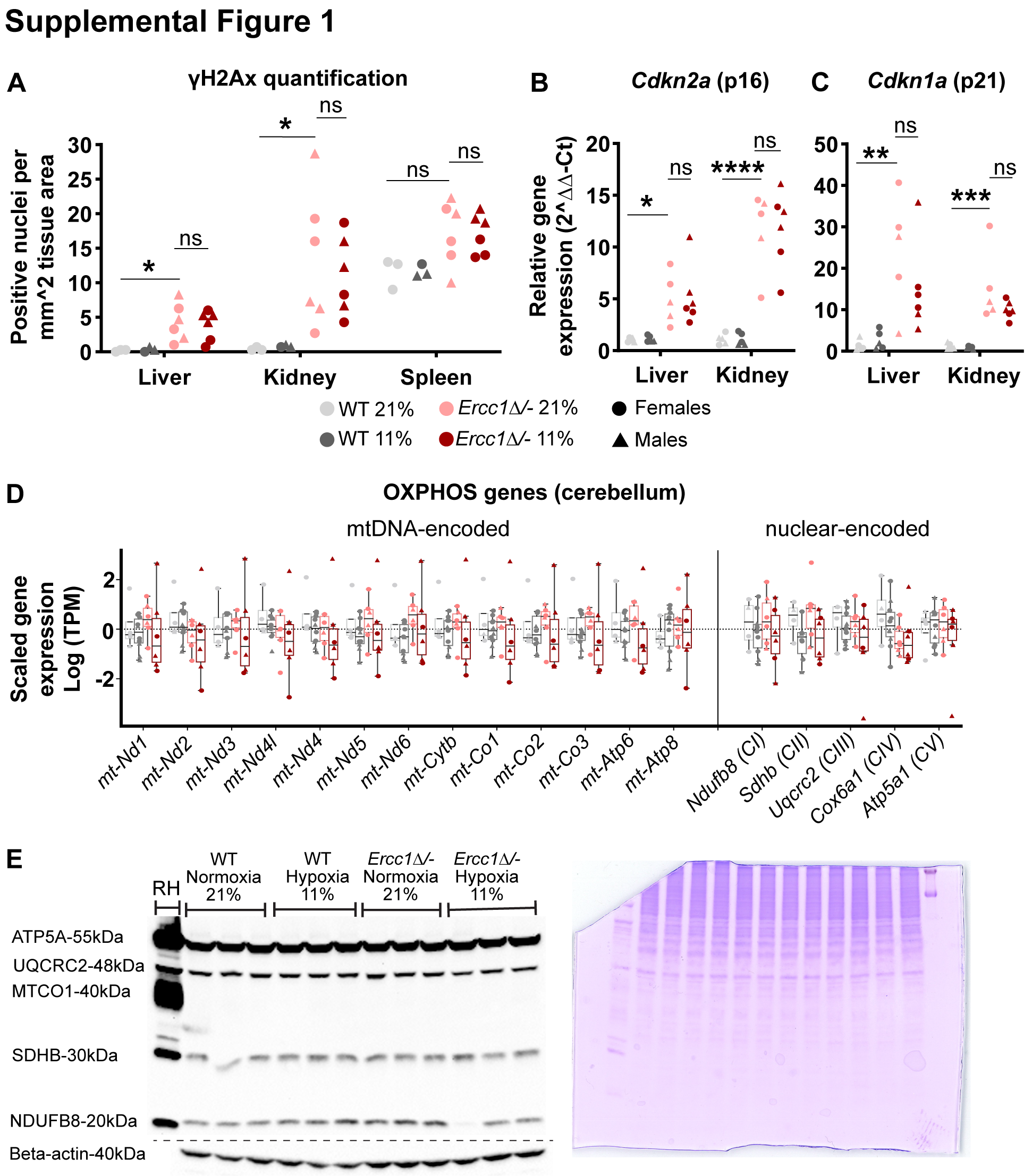

Supplement: S1 Fig — (A) Quantification of cells with ɤH2Ax foci at approximately 15 weeks of age (n = 3 per WT group, n = 6 per Ercc1 Δ/- group). Dunnett’s multiple comparisons test. S1 Data, Worksheet “gammaH2xIHC”; representative images in S2 Fig. (B, C) Relative gene expression of senescence markers at 14–19 weeks of age (n = 5–6 per group—dots plotted as mean of 2 technical replicates per sample, statistics calculated as nested analyses). Dunnett’s multiple comparisons test. S1 Data, Worksheet “qPCR”. (D) Relative expression of the 13 mt-mRNAs and 5 representative nuclear-encoded mRNAs in cerebellum at 15–19 weeks; n = 4, 10, 6, 6 as in Fig 2A–2F. Mouse basic metadata in S1 Data, Worksheet “RNAseq”. (E) Western blot of ETC subunits in forebrain at 14 to 15 weeks with accompanying beta-actin loading control and Coomassie blue gel stain; n = 3 per group. RH = purified rat heart mitochondria (positive control). Mouse metadata in S1 Data, Worksheet “westerns”. **** = p < 0.0001; *** = p < 0.001; ** = p < 0.01; * = p < 0.05; ns = not significant. (TIF) [file pbio.3002117.s002.tif]

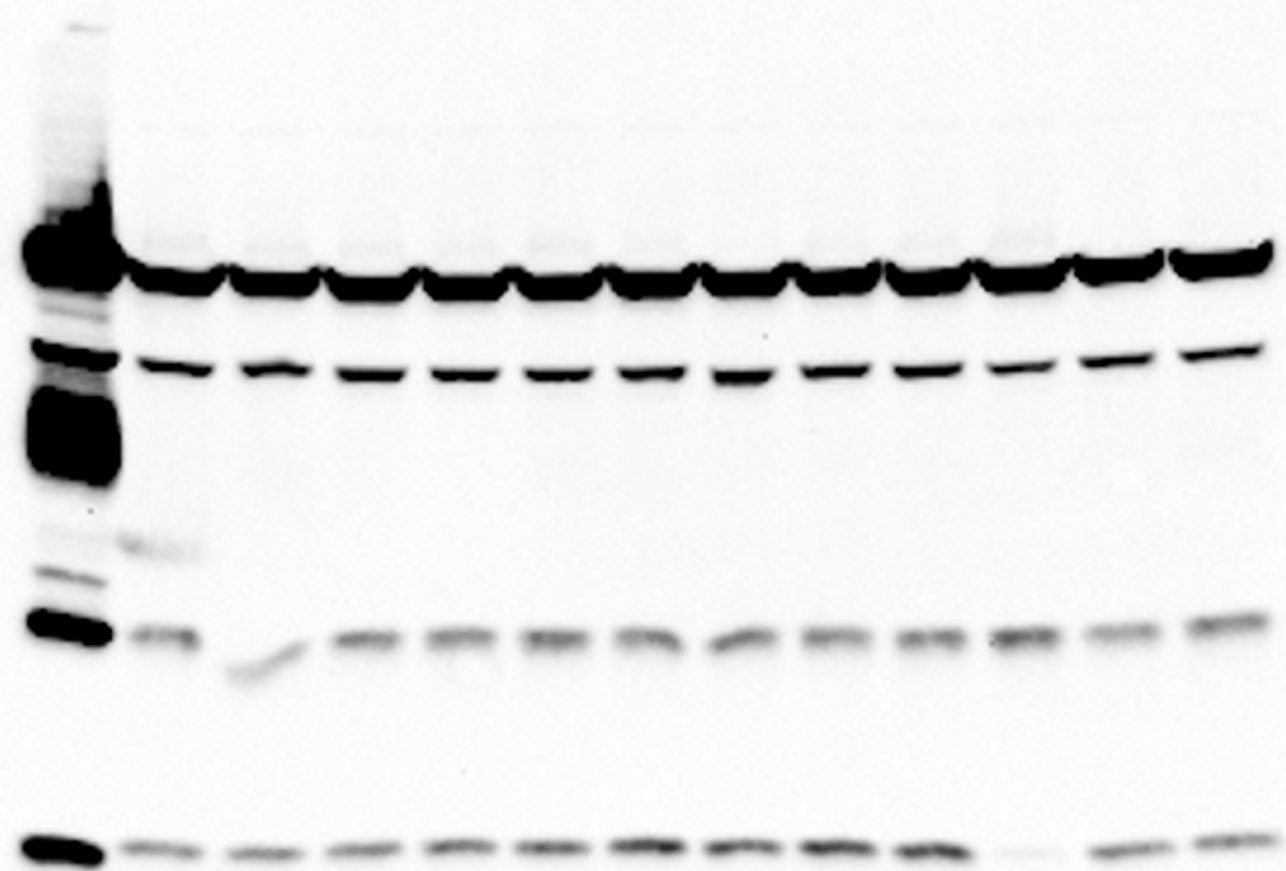

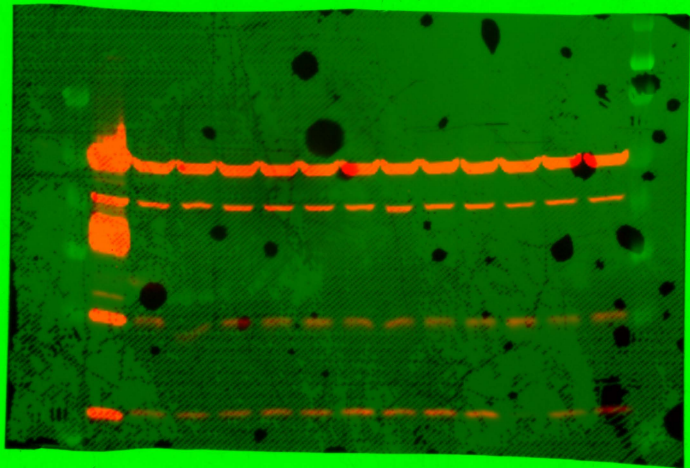

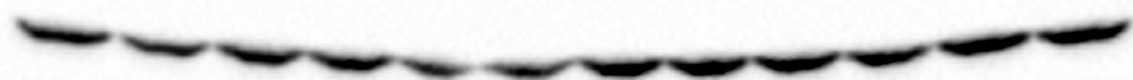

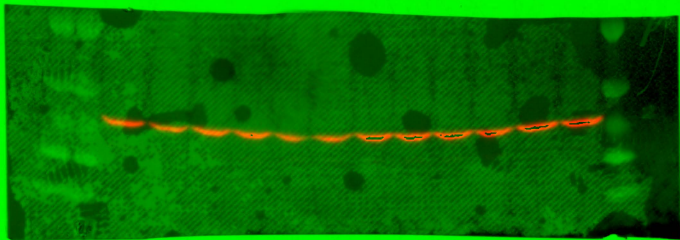

Oxphos Brain 7/14

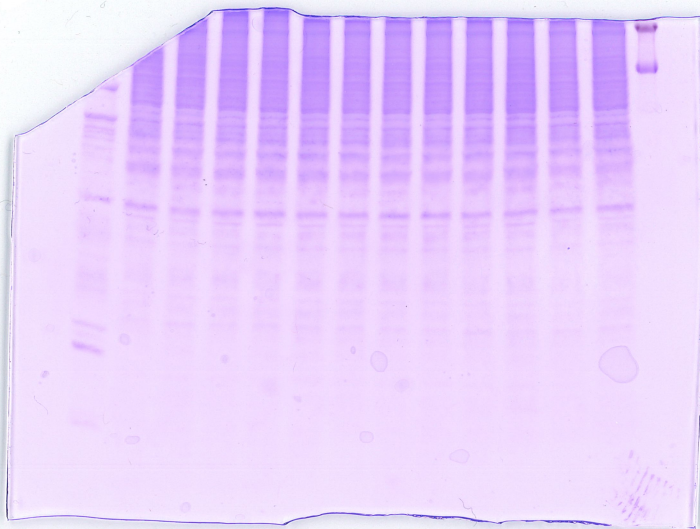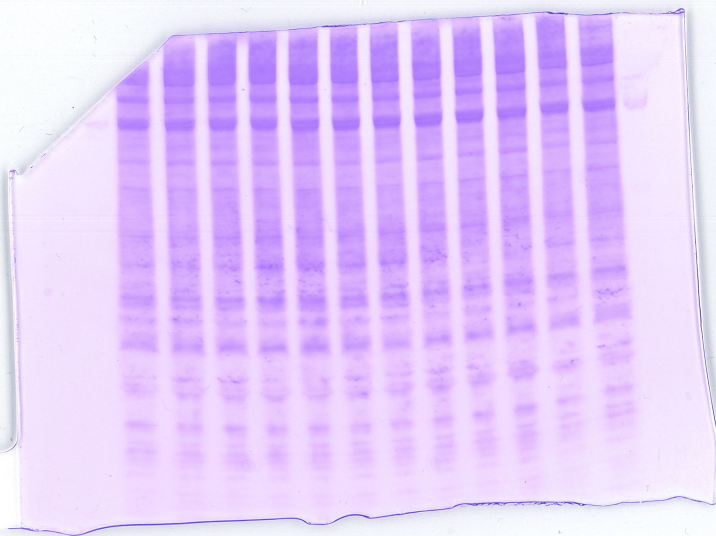

HIF1 $\alpha$  +  $\beta$ Actin Brain 7/14

Supplement: S1 Raw Images — (PDF) [file pbio.3002117.s008.pdf]
