# Supplementary material for: Hypoxia extends lifespan and neurological function in a mouse model of aging
Source: PLoS Biol. 2023 May 23;21(5):e3002117. doi: 10.1371/journal.pbio.3002117 (PMC10204955; doi:10.1371/journal.pbio.3002117)
Supplement: S2 Fig — (PDF) [file pbio.3002117.s003.pdf]

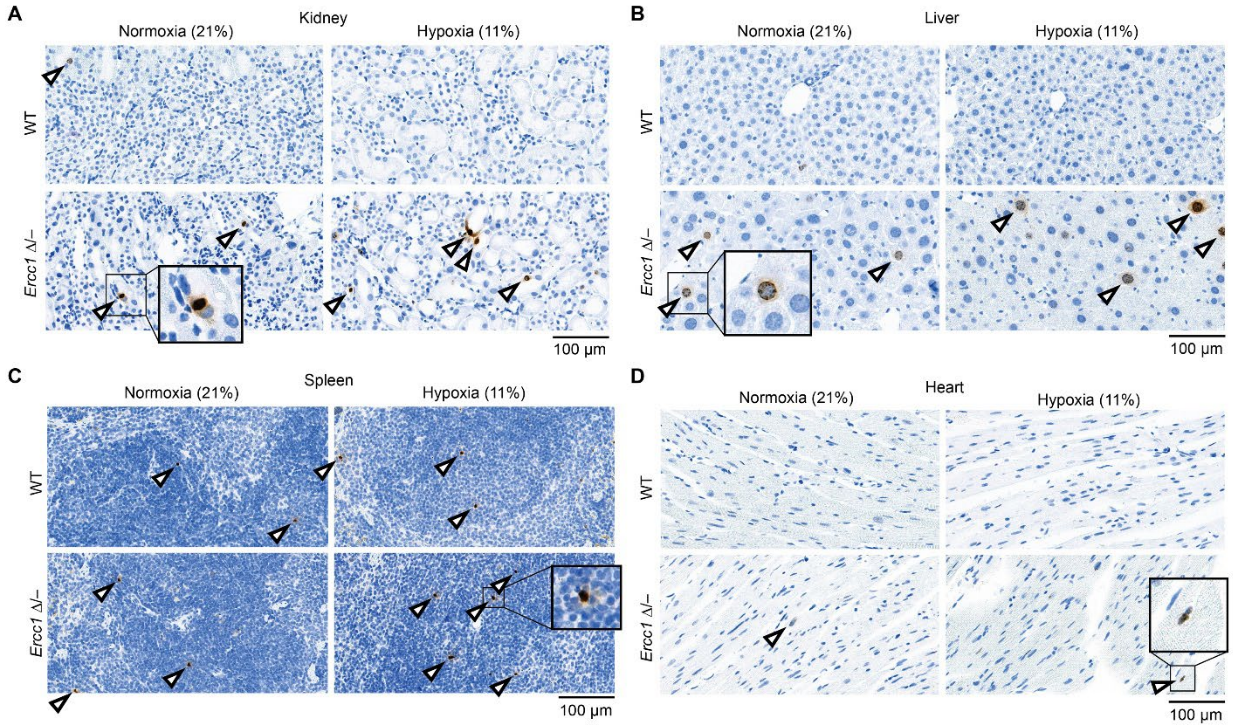

Supplemental Figure 2. Representative images of gamma-H2x IHC. (A-D) gamma-H2x IHC (brown) of kidney (A), liver (B), spleen (C), and heart (D). Magnification = 40x.
