# Supplementary material for: Hypoxia extends lifespan and neurological function in a mouse model of aging
Source: PLoS Biol. 2023 May 23;21(5):e3002117. doi: 10.1371/journal.pbio.3002117 (PMC10204955; doi:10.1371/journal.pbio.3002117)
Supplement: S1 Extended Methods — (DOCX) [file pbio.3002117.s007.docx]

**Extended Materials & Methods**

**Mice:** C57BL/6 *Ercc1* *+/-* and FVB *Ercc1 +/∆* mice were generously provided by the laboratory of Dr. Ingrid van der Pluijm and bred as initially described by Weeda et al in 1997^1^. Mice were weaned and genotyped 4 weeks after birth. Mice were housed 2 to 5 per cage, provided with food and water ad-libitum and a standard 12-hour dark-light cycle. Body weights were recorded weekly. Mice were euthanized when ataxia progressed to the point where severe immobility made them unable to acquire food or water. Euthanasia criteria were assessed by a central animal facilities technician not directly associated with this study, using American Veterinary Medical Association best practice. Three male mice were euthanized because of the development of severe and infected penile prolapse (2 male mice in normoxia at 15 weeks and 4 days, 1 male mouse in hypoxia at 25 weeks and 1 day). Because these mice were euthanized for reasons other than severe ataxia, they were excluded from the survival calculation; their inclusion would have accentuated the reported survival benefit of hypoxia treatment, not diminished it. Although *Ercc1 +/-* and *Ercc1 +/∆* heterozygotes have no discernible phenotype, they were not included in any analyses presented here and thus all wildtype mice are hybrid C57BL/6 – FVB *Ercc1 +/+*. All mouse studies were approved by the Subcommittee on Research Animal Care and the Institutional Animal Care and Use Committee of Massachusetts General Hospital under Protocol #2011N000077.

**Hypoxia exposure­:** All experiments were performed at sea-level. For hypoxia (11% oxygen), mice were placed in BioSpherix OxyCycler Model A84XOV (Parish, NY). This system enabled automated control and monitoring of the oxygen and carbon dioxide levels and allows us to house mice at the specified gas tension. N2 for dilution of air was supplied by liquid nitrogen tanks at approximately 235 psi (Med-Tech Gases, Billerica, MA). Soda lime (Sodasorb, Smiths Medical) (approximately 250g), was placed on the floor of each chamber to scavenge carbon dioxide (CO_2_) exhaled by the animals and replaced every 3 days. Temperature and humidity were maintained at 23–25°C and 30–70%, respectively. Mice were exposed to gas treatment continuously for 24 hours per day, 7 days a week. The chambers were briefly opened three times a week to weigh the mice, evaluate their neurological status, clean the cages, and add water and food.

Due to a sensor malfunction between November 15, 2020 and November 19, 2020, 6 of the *Ercc1* ∆/- mice assigned to continuous hypoxia and analyzed for survival and body weight were exposed to up to 4 days of normoxia between months 5 and 6 of life which might have had a detrimental effect on their survival (which would lead to underestimation of the reported survival benefit of continuous hypoxia).

**Accelerating rotarod test:** The amount of time mice were able to maintain their position on an accelerating rotating rod (Ugo Basile) was recorded. Mice were acclimated to the experimental room for at least 30 minutes before the start of the measurements. Rotarod parameters were as follows: acceleration of 5 rpm/m and a maximum speed of 40 rpm. If mice used their body to grasp the rod (rather than walking on it) for more than 10 s, this time was recorded as time of fall. Each mouse was tested three times.

**Hematocrit:** 30 to 50 uL of blood was removed from the tail vein and hematocrit was measured using the ABL800 Flex (Fisher Scientific, Waltham, MA).

**Peripheral smears for reticulocyte quantification:** Prior to sacrifice for histological studies as above, a small puncture was made in the mouse jugular vein and 35 uL of venous blood was collected into a 1.5 mL Eppendorf tube with 35 uL of 500 mM EDTA. The tubes were mixed by gentle inversion, and then 50 uL of the blood mixture was mixed with 50 uL of new methylene blue stain (Volu-Sol, Amazon). After mixing with gentle inversion, the tubes were incubated (room temperature, 15 min) and then peripheral blood smears were applied to glass slides per standard technique. A region of the peripheral blood smear with an appropriate (moderate) density of cells was identified (near the feathered edge, relatively high density of cells but without many overlapping or clumped cells, which would preclude accurate counting). Erythroid cells were identified based on their characteristic morphology. Reticulocytes were identified as erythroid cells with blue granules or filaments stained by the new methylene blue stain. Mature red blood cells (RBCs) were identified as erythroid cells without blue granules. A region with appropriate new methylene blue staining was identified (at least one reticulocyte was typically seen in a given 100x high-powered field). After a region with appropriate cell density and adequate staining was identified, a new 100x field for quantitation was randomly selected in order to reduce the risk of field selection bias. This random field was selected by blindly moving the field of view (100x, "high-power") 2-5 fields away from the field identified as an adequate region and then counting this new field. Both mature RBCs and reticulocytes were manually counted by 100x oil-immersion light microscopy. At least 1000 cells were counted for each slide. The total number of cells counted and the % reticulocytes were recorded.

**Histology:** Mice were placed and maintained under deep anesthesia with 5% isoflurane in oxygen for the extent of the perfusion. The chest cavity was opened and a catheter was placed in the left ventricle, after which the right atrium was cut. Mice were perfused with heparin in PBS, followed by 20 to 30 mL of 10% neutral buffered formalin (NBF) at room temperature. Organs were dissected from perfused mice in the following order: brain, liver, spleen, kidney, heart. Organs were immediately post-fixed in 10% NBF for 24 hours and then delivered to the Specialized Histopathology core lab of Dana Farber/Harvard Cancer Center (Longwood).

For histology, tissue was processed (Leica ASP300 S automated vacuum tissue processor; Small Program), embedded (Leica EG1150), and sectioned (Leica 2255 microtome) at 5 microns. Hematoxylin & eosin (H&E) staining was performed with a Leica Spectra ST. Immunohistochemistry (IHC) was performed on a Leica Bond III automated staining platform using a Leica Biosystems Refine Detection Kit (#DS9800). Antibodies were used as follows (all antigen retrieval was in citrate for 30 minutes): phospho-histone H2A.X (Cell Signaling Technology, #9718, dilution 1:750). Images were scanned at 40X into the Aperio electronic slide management system and analyzed.

**γH2Ax quantification:** Tissue was processed for IHC as described above. For each tissue, a cell was counted as positive for γH2Ax if its nucleus was stained with at least moderate intensity (brown) signal. γH2Ax-positive cells were counted in twelve different medium power (500 x 500 micron, 20X) fields, sampling all available areas of the tissue section. Counts were normalized to a square millimeter of tissue. Representative images were captured.

**Bulk RNAseq of the cerebellum:** Birth cohorts of mice were euthanized as described above at the time when the *Ercc1 ∆/-* normoxic mice of that cohort reached euthanasia criteria, between 15 and 19 weeks. The cerebellum was carefully dissected from the remainder of the brain and snap frozen at -80^o^C until further processing. RNA extraction, library preparation and sequencing were performed by Genewiz (now Azenta, South Plainfield, NJ). 27 cerebella were submitted for sequencing; 1 of the 5 wildtype normoxic samples was a distinct outlier on principal component analysis and the calculated Cook’s distance of several genes and so was excluded from downstream analysis.

After RNA extraction with polyA selection, unstranded library preparation was performed for 150 bp sequencing. Samples were distributed across 2 lanes with 350 million raw paired-end reads per lane and sequencing was performed on Illumina HiSeq platform. Data was provided in FASTQ format and initial quality control was run through FASTQC^2^, demonstrating high sequence quality scores across all samples. Gene expression estimates were calculated using the following pipeline:

Read alignment was performed with the STAR aligner version 2.7.5a^3^ using ‘—twopassMode Basic’ with the mm10 genome downloaded from the UCSC Genome Browser website (https://hgdownload.soe.ucsc.edu/goldenPath/mm10/bigZips/chromFa.tar.gz). Any reads aligning with equal mapping quality to a NUMT region on a nuclear chromosome and chrM were assigned to chrM using a custom script (prioritize_chrM_over_NUMTS.py). Duplicate reads were removed using the Picard MarkDuplicates utility (version 2.21.9)^4^ included in the Genome Analysis Toolkit (GATK, version 4.1.5.0)^5^. Finally, reads were assigned to genes in the mouse GENCODE version M24 genome annotation using the featureCounts program included in the Subread package (version 2.0.1)^6^ with the options: -t exon -p -M --primary. The featureCounts output for each sample was used to assemble an AnnData object (version 0.7.5), part of the Scanpy ecosystem (version 1.8.1)^7^, in Python (version 3.7.12) for downstream analysis. The raw counts were converted into units of transcripts per million (TPM) using the length values provided in the featureCounts output, which are computed as the number of exonic DNA bases in each gene.

The log(TPM + 1) values were used as input to the scanpy.pp.highly_variable_genes function. We selected genes with mean log(TPM + 1) values greater than 0.5 and less than 7, and with normalized dispersion greater than 0.75, yielding a set of 4,798 genes that contain most of the variable signal in the data. Mindful that the global mean used in this selection is an average of gene expression across all of our experimental conditions and also across all cell types in the mouse cerebellum, we set our minimum gene expression threshold high enough to exclude genes that are off or expressed at vanishingly low levels, but low enough to still capture genes that might be robustly expressed in a cell type- or condition-specific manner. We chose our dispersion threshold to include all genes that met the gene expression criteria that also have dispersion across our samples that is about the same as *Ercc1* or greater. We z-score scaled the log(TPM) values for each gene using the scanpy.pp.scale function with max_value=10. These scaled values were used as input to PCA, using scanpy.tl.pca with solver=’arpack’.

**Differential gene expression testing**: Differential gene expression (DE) was performed on the raw count data for our set of 4,798 highly variable genes with the DESeq2 package (version 1.30.1)^8^ for *Ercc1 Δ/-* in normoxia vs wildtype in normoxia and *Ercc1 Δ/-* in hypoxia vs *Ercc1 Δ/-* in normoxia. A false discovery rate of 0.01 and an absolute value of the log2 fold change of 1.25 was used to identify differentially expressed genes in pairwise comparisons. We used Enrichr^9^ through the GSEAPY package (version 0.10.3)^10^ to test our DE gene lists against the GO_Biological_Process_2018, KEGG_2019_Mouse, and MGI_Mammalian_Phenotype_Level_4_2019 gene set databases. We highlight a set of 305 innate immune genes, defined as the set of genes associated with the top three most significant GO terms from our Enrichr analysis of the upgoing differentially expressed genes in the *Ercc1 ∆/-* vs WT normoxia comparison (i.e. GO:0019221, GO:0071357, and GO:0060337).

**Computational detection of DNA damage signature in RNA-seq data:** We tested whether there was a difference in the distribution of gene length for differentially expressed genes going up or going down in *Ercc1 Δ/-* compared to wild type. For two DE comparisons, *Ercc1 Δ/-* in normoxia vs wildtype in normoxia and *Ercc1 Δ/-* in hypoxia vs wildtype in normoxia, we split the 4,798 genes included in the DE analysis into three categories: DE genes going up in the *Ercc1 Δ/-* mice, DE genes going down in the *Ercc1 Δ/-* mice, and non-DE genes. We extracted chromosomal coordinates for each gene from the GENCODE version M24 annotation, calculated the gene lengths in total DNA base pairs for the genes in each of the three categories, and generated empirical cumulative distribution functions of gene length for each category. We then tested whether the eCDF for the upgoing DE genes was enriched for shorter genes compared to the non-DE eCDF, and whether the eCDF for the downgoing DE genes was enriched for longer genes compared to the non-DE eCDF using one-sided 2-sample Kolmogorov-Smirnov tests, implemented in the scipy.stats.ks_2samp function in SciPy (version 1.7.0)^11^.

**Gene expression via qPCR:** RNA was extracted with the RNEasy extraction kit (Qiagen) per manufacturer instructions, eluted in water and quantified and assessed for purity with Nanodrop. 1 ug of RNA per sample was converted to cDNA with SuperScript III First Strand Synthesis Super Mix for qRT-PCR (ThermoFisher) per manufacturer protocol. qPCR was performed with Taqman (ThermoFisher) using the following probes: Beta-actin=Mm01205647; CDKN1a=p21=Mm04205640; CDKN2a=p16=Mm00494449; PCR reaction was run on 7500 Fast Real Time PCR System (ThermoFisher) for 40 cycles alternating between 95C and 60C. Relative gene expression is reported as 2^-(delta/delta Ct) with wild-type normoxia beta-actin as the reference condition. Five to six biological replicates of each condition were performed for all genes reported with two technical replicates per biological replicate. The average of the two technical replicates corresponds to the dot in the dot plot, but significance was calculated using nested analyses considering both technical replicates.

**Western immunoblots:** Tissue was homogenized on ice in RIPA buffer with EDTA (Boston BioProducts) and 1% protease-phosphatase inhibitor (Cell Signaling Technology), protein was quantified by BCA (ThermoFisher), mixed in 6X SDS sample buffer (Boston BioProducts), denatured by boiling at 95C for 5 minutes (resulting in expected loss of heat-labile Complex IV), and then frozen at -80^o^C until further analysis.

OXPHOS Cocktail: Protein was loaded at 10 ug per lane on a 12% Tris-Glycine gel (rat heart mitochondrial protein extract was added in lane 1 as a reference), blocked in 5% milk in TBST. Total rodent OXPHOS cocktail primary antibody (ab110413, Abcam) was added at 1:250 in PBS with 1% milk; anti-mouse secondary antibody was added at 1:10,000 in 5% milk in TBST.

Beta-actin: Protein was loaded at 20 ug protein per lane on a 4-12% Tris-Glycine gel, block in 5% milk in TBST. Beta-actin primary antibody (4967S, Cell Signaling Technology) was added at 1:1000 in 5% milk in TBST, anti-rabbit secondary antibody was added at 1:10,000 in 5% milk in TBST.

After ECL exposure, membrane was developed with Biorad ChemiDoc MP using ImageLab 5.2.1 software.

**mtDNA sequencing:** DNA was extracted with the DNEasy extraction kit (Qiagen) per manufacturer instructions, eluted in water and quantified and assessed for purity with Nanodrop. mtDNA was amplified in two fragments following the protocol of Ma et al in 2018^12^: “fragment 1: F-3222 GGATCCTACTCTCTACAAAC and R- 11432 TAGTTTGCCGCGTTGGGTGG; fragment 2: F-11271 CTACCCCCTTCAATCAATCT and R- 3335 CCGGTTTGTTTCTGCTAGGG.” Primers were purchased from IDT.

For two fragment amplification, the TAKARA LA Taq kit (Clontech) was used with 200ng of DNA input per sample. “Two fragment PCR reaction was performed under the following conditions: one cycle at 94°C for 5 min, then 30 cycles at 94°C for 30 sec, 56°C for 20 sec and 68^o^C for 7 min, and followed by one cycle at 68°C for 3 min.”

After amplification, 10 uL of DNA was run on agarose gel treated with ethidium bromide to confirm the presence of a single ~8kb band. The remaining amplified DNA (40 uL) was purified with Ampure XP (Beckman) beads using 1.8 uL beads’ solution per 1uL PCR reaction and following manufacturer’s protocol. Purified amplicons were then sent to Genewiz (now Azenta) for sequencing.

The amplified mitochondrial genomic DNA was sequenced with 300 bp paired end reads on the Illumina MiSeq platform. Data was provided in FASTQ format and initial quality control was run through FASTQC^2^. First, the reads were used to generate sample-specific mitochondrial genome reference sequences. Briefly, the reads were aligned to two versions of the chrM reference from mm10 using Bowtie2 (version 2.4.1)^13^ to account for the circular nature of the chrM genome; one chrM reference sequence was the regular linearized chrM sequence, and the other was shifted so that the usual breakpoint was in the middle of the linearized chrM sequence. The mapping coordinates for the reads on the shifted chrM were unshifted so that they corresponded to the mm10 chrM breakpoint, and each read was assigned a single alignment position based on which alignment had the best mapping quality. Then we used a custom script based on the SAMTools pileup command (samtools version 1.12)^14^ to identify positions where the majority of reads differed from the mm10 reference sequence and wrote these into a custom chrM fasta file. This custom reference improves our read mapping by avoiding mismatches that are due simply to inherited variants in the mouse line, and therefore prioritizes the calling of somatic mutations. We then masked regions of nuclear mitochondrial DNA (NUMTs) in the custom references^15^ by generating synthetic sequencing reads using ART (version 2016.06.05)^16^, mapping them to the nuclear genome using Bowtie2, calling regions with significant mapping using the MACS2 peak caller (version 2.2.7.1)^17^, and masking those regions using the BEDTools maskfasta command (version 2.29.2)^18^.

After generating the sample-specific references, we proceeded to align the reads. Trimmomatic (version 0.39)^19^ was used to remove Illumina adapter sequences from the ends of reads with the options ILLUMINACLIP:NexteraPE-PE.fa:2:30:10:1:true, TRAILING:3, SLIDINGWINDOW:4:10, and MINLEN:20. Then the reads were aligned with Bowtie2 with the option -X 2000, and keeping only reads mapped in proper pairs with a mapping quality score of at least 10. Next, we used ABRA2 (version 2.23)^20^ to do local realignment of reads, and removed duplicates using the Picard MarkDuplicates utility (version 2.21.9)^4^ included in the Genome Analysis Toolkit (GATK, version 4.1.5.0)^5^. We identified SNVs using a custom script based on the SAMTools pileup command (samtools version 1.12)^14^, and assessed the potential impact of the variants with the ENSEMBL Variant Effect Predictor (VEP) (version 102.0)^21^.

**Statistics:** The following statistics were calculated using GraphPad Prism (Version 9): for survival analyses, the Mantel-Cox and Gehan-Brislow-Wilcoxon tests; for neurologic performance, quantitation of histological data and qPCR, one-way ANOVA was performed followed by individual pair-wise comparisons adjusting for multiple hypothesis testing; a p-value of 0.05 was considered significant. For differential gene expression, statistical analyses were performed using the DESeq2 package with the threshold for significance applied as indicated in the text. The Wang-Allison test (two-tailed to be conservative) of maximal survival was conducted in Python using the scipy.stats.boschloo_exact function from SciPy (version 1.7.0)^11^. The cumulative density functions were calculated and plotted in Python using the seaborn.ecdfplot function (version 0.11.1)^22^, and the corresponding Kolmogorov-Smirnov D-statistics were calculated in Python using the scipy.stats.ks_2samp function in SciPy (version 1.7.0)^11^. Tests of differential OXPHOS gene expression were calculated in Python using the functions smf.ols and sm.stats.anova_lm from statsmodels (version 0.11.1)^22^.

**Methods References:**

1 Weeda, G. *et al.* Disruption of mouse ERCC1 results in a novel repair syndrome with growth failure, nuclear abnormalities and senescence. *Curr Biol* **7**, 427-439, doi:10.1016/s0960-9822(06)00190-4 (1997).

2 Andrews, S. *A Quality Control Tool for High Throughput Sequence Data [Online]*, <<http://www.bioinformatics.babraham.ac.uk/projects/fastqc/>> (2010).

3 Dobin, A. *et al.* STAR: ultrafast universal RNA-seq aligner. *Bioinformatics* **29**, 15-21, doi:10.1093/bioinformatics/bts635 (2013).

4 Repository, B. I. G. *2019*, <<https://broadinstitute.github.io/picard/>> (Picard Toolkit).

5 McKenna, A. *et al.* The Genome Analysis Toolkit: a MapReduce framework for analyzing next-generation DNA sequencing data. *Genome Res* **20**, 1297-1303, doi:10.1101/gr.107524.110 (2010).

6 Liao, Y., Smyth, G. K. & Shi, W. featureCounts: an efficient general purpose program for assigning sequence reads to genomic features. *Bioinformatics* **30**, 923-930, doi:10.1093/bioinformatics/btt656 (2014).

7 Wolf, F. A., Angerer, P. & Theis, F. J. SCANPY: large-scale single-cell gene expression data analysis. *Genome Biol* **19**, 15, doi:10.1186/s13059-017-1382-0 (2018).

8 Love, M. I., Huber, W. & Anders, S. Moderated estimation of fold change and dispersion for RNA-seq data with DESeq2. *Genome Biol* **15**, 550, doi:10.1186/s13059-014-0550-8 (2014).

9 Kuleshov, M. V. *et al.* Enrichr: a comprehensive gene set enrichment analysis web server 2016 update. *Nucleic Acids Res* **44**, W90-97, doi:10.1093/nar/gkw377 (2016).

10 Fang, Z. GSEApy. GitHub Repository. (2021).

11 Virtanen, P. *et al.* SciPy 1.0: fundamental algorithms for scientific computing in Python. *Nat Methods* **17**, 261-272, doi:10.1038/s41592-019-0686-2 (2020).

12 Ma, H. *et al.* Germline and somatic mtDNA mutations in mouse aging. *PLoS One* **13**, e0201304, doi:10.1371/journal.pone.0201304 (2018).

13 Langmead, B. & Salzberg, S. L. Fast gapped-read alignment with Bowtie 2. *Nat Methods* **9**, 357-359, doi:10.1038/nmeth.1923 (2012).

14 Li, H. *et al.* The Sequence Alignment/Map format and SAMtools. *Bioinformatics* **25**, 2078-2079, doi:10.1093/bioinformatics/btp352 (2009).

15 Lareau, C. A. *et al.* Massively parallel single-cell mitochondrial DNA genotyping and chromatin profiling. *Nat Biotechnol* **39**, 451-461, doi:10.1038/s41587-020-0645-6 (2021).

16 Huang, W., Li, L., Myers, J. R. & Marth, G. T. ART: a next-generation sequencing read simulator. *Bioinformatics* **28**, 593-594, doi:10.1093/bioinformatics/btr708 (2012).

17 Zhang, Y. *et al.* Model-based analysis of ChIP-Seq (MACS). *Genome Biol* **9**, R137, doi:10.1186/gb-2008-9-9-r137 (2008).

18 Quinlan, A. R. & Hall, I. M. BEDTools: a flexible suite of utilities for comparing genomic features. *Bioinformatics* **26**, 841-842, doi:10.1093/bioinformatics/btq033 (2010).

19 Bolger, A. M., Lohse, M. & Usadel, B. Trimmomatic: a flexible trimmer for Illumina sequence data. *Bioinformatics* **30**, 2114-2120, doi:10.1093/bioinformatics/btu170 (2014).

20 Mose, L. E., Perou, C. M. & Parker, J. S. Improved indel detection in DNA and RNA via realignment with ABRA2. *Bioinformatics* **35**, 2966-2973, doi:10.1093/bioinformatics/btz033 (2019).

21 McLaren, W. *et al.* The Ensembl Variant Effect Predictor. *Genome Biol* **17**, 122, doi:10.1186/s13059-016-0974-4 (2016).

22 Seabold, S., and Josef Perktold. Statsmodels: Econometric and Statistical Modeling with Python. 92-96 (2010).
